# Mitochondrial Genes Cause Nuclear Mischief

**DOI:** 10.1371/journal.pbio.0020316

**Published:** 2004-09-07

While the nucleus of a cell may be its command headquarters, mitochondria are equally vital—they are the power plants of the cell, and without them all cellular activity would quickly and irrevocably come to a halt. Testifying to their origins as once free-living bacteria, mitochondria have their own DNA, comprising 37 genes in humans on a single circular chromosome. Whether they invaded their ancestral hosts as parasites or were captured as subcellular collaborators, they have long since left their independent ways behind. Their meager complement of genes is far fewer than is needed to produce these complex organelles; it is clear from analyzing the nuclear genome that most of the mitochondria's presumed ancestral genes have been taken into the cell's nucleus, where they are under the strict control of their host.

The transplanted mitochondrial genes have been faithfully doing their job under new management since they were first appropriated, probably hundreds of millions of years ago. However, not all of their DNA descendants have continued to make themselves so useful. For, in addition to many of the mitochondria's original genes, the human genome houses over 200 mitochondrial genetic fragments, useless pieces of code whose only remaining function is to be replicated generation after generation.

Detritus from other sources is even more common within the genome, and most of it seems to be harmless. But in this issue, Ricchetti and colleagues show that mitochondrial fragments may not be quite so benign. They have continued to invade the human genome, even into the present day, and a large proportion of them take up residence within nuclear genes, possibly disrupting them and causing human diseases.

Scanning the entire human genome, Ricchetti and colleagues found a total of 211 nuclear sequences of mitochondrial origin (NUMTs). Of these, they selected 42, which appeared to be the most recent integrations, for detailed study. Only 14 of them were also found in DNA from our closest relatives, chimpanzees, indicating that the rest arose after the human–chimp split approximately 5 million years ago. While 35 of the 42 were found in all humans tested, the rest were not, suggesting a still more recent origin for these among human populations.

The authors also made two surprising discoveries about the location of these human-specific NUMTs. They were not evenly distributed across the entire genome; instead, for reasons that are unclear, there were a disproportionate number of them on two chromosomes—the Y chromosome, present only in males, and number 18. Furthermore, NUMTs were not randomly scattered among all the DNA of the chromosomes. Rather, they were much less likely to be found in non-coding “junk” DNA and much more likely to have inserted themselves within highly active genes. This phenomenon is likely to be related to the mechanism by which a NUMT enters the chromosome—it relies on the machinery that repairs breaks in the DNA, and these breaks are more common in genes that are frequently transcribed. Such insertions can cause disease, as shown by the recent discovery of a hemophilia patient with a NUMT interrupting his clotting factor gene.

Much remains to be learned about the functional and temporal dynamics of NUMT insertions, but their potential for harm suggests that many NUMTS, unlike much of the rest of the flotsam that litters our genome, may be selected against quickly. Combined with their differential distribution among human ethnic groups, this may make them valuable markers for tracking both long- and short-term trends in human evolution and migration.

